# Variations in sensory eye dominance along the horizontal meridian

**DOI:** 10.1167/jov.25.8.1

**Published:** 2025-07-01

**Authors:** Chris L. E. Paffen

**Affiliations:** 1 Helmholtz Institute & Experimental Psychology, Utrecht University, the Netherlands

**Keywords:** eye dominance, sensory eye dominance, binocular vision, binocular rivalry

## Abstract

Sensory eye dominance refers to the dominance of one eye's input over the other during interocular conflict, such that, when discrepant images are presented dichoptically, one eye's image will dominate perception. This study focuses on how sensory eye dominance varies across visual space. Although some characteristics of variations in sensory eye dominance across visual space have been described before, results so far are largely conflicting. Here I argue that this conflict is caused by the fact that different studies used different methods to assess sensory eye dominance, combined with using a wide range of eccentricities. To systematically and continuously describe sensory eye dominance across the visual field, I used a novel method—tracking Continuous Flash Suppression—in which a visual target presented to a single eye moved across the horizontal meridian while being in constant competition with a dynamic mask presented to the other eye. Eye dominance across the visual field could be described and quantified using three factors: (1) a generic preference for the nasal visual field in combination with (2) an observer-dependent general bias for using the left, right, or neither eye. On top of these, some observers had (3) idiosyncratic biases in local sensory eye dominance. I argue that, while idiosynchratic local biases within an observer probably stem from optical, retinal, or cortical imbalances, the observed nasal advantage is functional: it allows to bias the interocular competition to fixated, partly occluded distant objects of interest.

## Introduction

Eye dominance is generally defined as the preference for using the left or right eye for executing a specific visual task, such as using a monocular (Ooi & He, 2020). One way to assess eye dominance is by using the hole-in-the-card test (Durand & Gould, 1910). In this test, an observer extends one arm and looks through a cylinder at a distant object. Next, the left and right eye are consecutively closed. When the dominant eye is closed, the distant object will be—visually—displaced with respect to the cylinder; closing the nondominant eye on the other hand will not displace the object. The hole-in-the-card test can be classified as falling under sighting dominance (Coren & Kaplan, 1973), which, next to acuity eye dominance and sensory eye dominance, is one of the three types of eye dominance that are generally distinguished. The second type of eye dominance, acuity eye dominance, simply refers to a difference in performance between the eyes on an acuity test such as the Snellen chart. The third type of eye dominance is sensory eye dominance (SED), which refers to the eye dominating perception when discrepant images are presented to overlapping retinal locations (e.g., Leat & Woodhouse, 1983). Such a situation generally leads to binocular rivalry, during which the discrepant images compete for perceptual dominance (Blake & Wilson, 2010). Over the years, it has repeatedly been investigated whether the three types of eye dominance are related. Although one study found a relation (Handa et al., 2004), most studies found no or a weak relation between the three types (Coren & Kaplan, 1973; Ding, Naber, Gayet, Van der Stigchel, & Paffen, 2018; Washburn, Faison, & Scott, 1934; Xu, He, & Ooi, 2011; Yang, Blake, & McDonald II, 2010).

Although a naive idea would hold that eye dominance is constant over the visual field, research has shown that both sighting (Carey & Hutchinson, 2013; Khan & Crawford, 2001) and sensory eye dominance (Chen & He, 2003; Crovitz & Lipscomb, 1963; Dieter, Sy, & Blake, 2017a; Dieter, Sy, & Blake, 2017b; Fahle, 1987; Kaushall, 1975; Leat & Woodhouse, 1983; Mustonen, Nuutinen, Vainio, & Häkkinen, 2018; Sahakian, Paffen, Van der Stigchel, & Gayet, 2022; Stanley, Carter, & Forte, 2011; Stanley, Forte, & Carter, 2019; Xu et al., 2011) vary across the visual field. Dieter et al. (2017a), for example, used binocular rivalry to show that an image presented to the left eye might dominate perception at location X, while an image presented to the right eye might dominate perception at location Y. On top of this, idiosyncratic individual differences between observers have been reported, where the pattern of local SED varies between observers (Dieter et al., 2017b; Stanley et al., 2011; Stanley et al., 2019; Xu et al., 2011).

**Figure 1.**
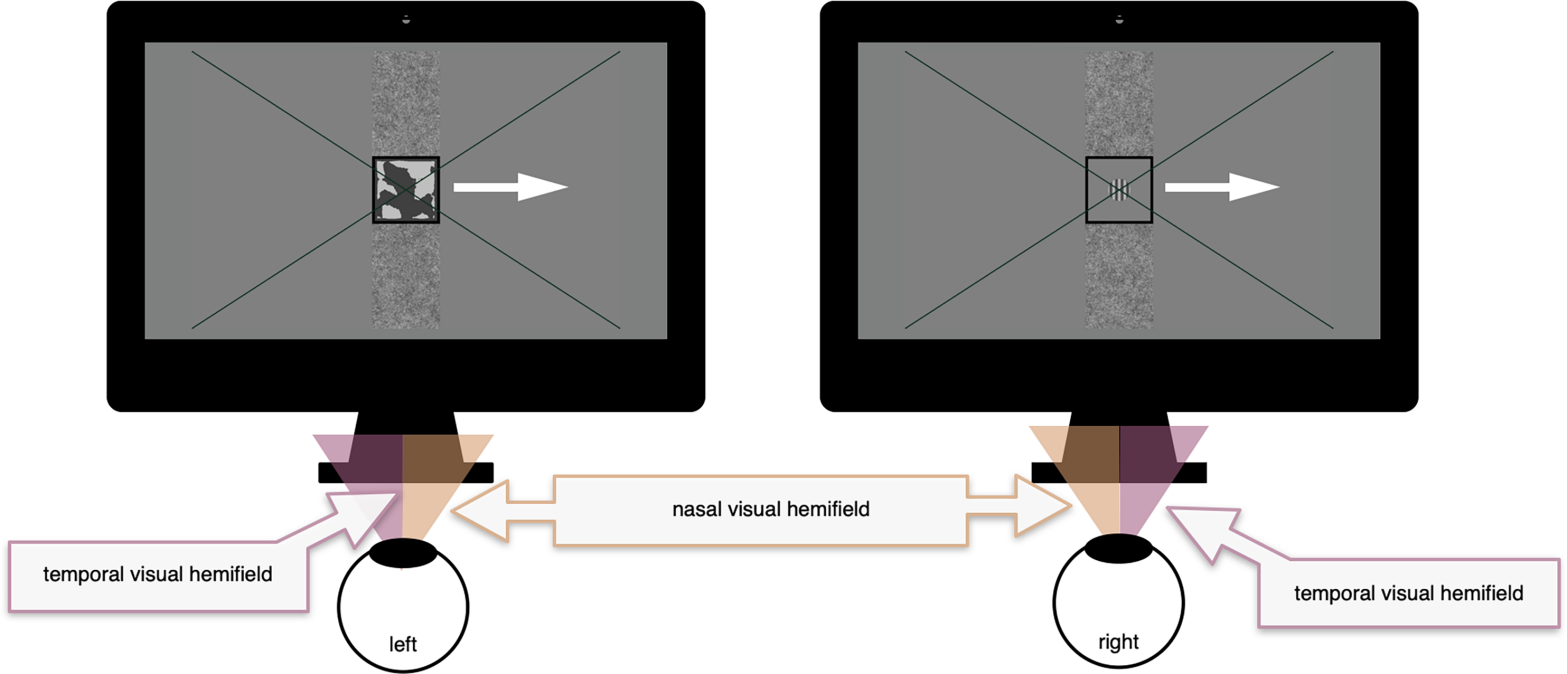
The stimulus used in the experiments. In the bCFS experiment, target and mask were presented at −10°, 0°, or 10° VA from fixation (at overlapping retinal locations for the left and right eye). The target would gradually increase in intensity until it was detected by the observer. In the tCFS experiment, target and mask would move in tandem across the screen: from 0° to –10°, to 10°, to 0° VA from fixation or from 0° to 10°, to –10° to 0° VA. The observer fixated the green cross and adjusted the intensity of the target continuously.

Whereas Dieter et al. (2017a) reported large differences between observers in local SED, other research also highlights generic aspects of sensory eye dominance varying across the visual field. Sahakian et al. (2022), for example, analyzed multiple datasets applying breaking Continuous Flash Suppression (bCFS). In this method, a target gradually increasing in intensity is presented to one eye, while a high-contrast dynamic mask is presented to the other eye (Jiang, Costello, Fang, Huang, & He, 2006). At a certain intensity, the target “breaks” suppression and becomes visible. Sahakian and colleagues showed that a nasal hemifield bias is observed when targets are presented in the visual periphery (e.g., a couple of degrees VA left or right of fixation). That is, when the target is presented to the nasal hemifield of one eye (and by definition, the mask is presented to the temporal hemifield of the other eye; see Figure 1) reaction times to the target are shorter than when the target is presented to the temporal hemifield (with the mask now presented to the nasal hemifield). Importantly, this result implies that eye dominance varies consistently across space: when the target is presented to the nasal hemifield of the *left* eye, it is presented at the *right* side of fixation; when the target is presented to the nasal hemifield of the *right* eye, it is presented to the *left* side of fixation. Thus, when a target is presented to the right side of fixation, sensory eye dominance (at that location) is higher for the left eye than for the right eye; when the target is presented to the left side of fixation, sensory dominance is higher for the right eye than for the left eye.

Sahakian et al. (2022) were not the first to report variations in SED across the visual field. Inspection of previous studies paints a complicated picture: while some studies’ results are in line with those of Sahakian and report a nasal preference (Chen & He, 2003; Kaushall, 1975; Mustonen et al., 2018), others reported a temporal preference (Crovitz & Lipscomb, 1963; Fahle, 1987; Stanley et al., 2011; Stanley et al., 2019), or did not find a preference for either (Dieter et al., 2017b).^1^ The different outcomes of these studies are likely due to the different paradigms used (see below) and the different stimulus parameters (e.g., eccentricities varying from less than 1° VA (Stanley et al., 2019) to 33° VA (Fahle, 1987). With regard to the different paradigms to evoke interocular conflict, studies generally use(d) one of three methods: the aforementioned methods of continuous tracking of the percept during prolonged interocular conflict (i.e., binocular rivalry) and detection of a target suppressed by breaking continuous flash suppression (i.e., bCFS), as well as reporting the first percept of discrepant monocular images (onset rivalry). It is known that these three methods can lead to different conclusions regarding which eye is dominant in SED. Ding et al. (2018) for example, found that the dominant eye as assessed by onset rivalry correlated with that as assessed by binocular rivalry, but not with that determined by breaking continuous flash suppression.

In this study, I seek to find a quantitative description of SED across the visual field, thereby possibly reconciling the results that point to idiosyncratic patterns of SED within observers with results showing generic visual field anisotropies (i.e., a hemifield bias). Keeping in mind the fact that different methods evoking interocular conflict can lead to different conclusions regarding which eye is dominant, I limit the method here to a variant of breaking continuous flash suppression. The reasons for choosing a variant of bCFS are twofold: (1) the method has consistently revealed a nasal visual field advantage in seven independent datasets,^2^ and (2) it provides a quick way to assess SED: for example, as one trial evoking binocular rivalry takes up to one minute, a bCFS trial only lasts several seconds. To use bCFS even more efficiently (i.e., to probe SED across the visual field), I adapted a method from Alais, Coorey, Blake, and Davidson (2024) to evoke interocular conflict continuously. In Alais et al's method, an observer not only indicates when a target increasing in intensity becomes visible during CFS; the observer additionally indicates when it becomes invisible when the intensity decreases (thus the stimulus waxes and wanes into awareness). Here I adapted Alais’ method—tracking continuous flash suppression, or tCFS—to make the target (and mask) move over the horizontal axis of the visual field.^3^ This method allowed me to measure (local) eye dominance continuously, thereby providing a much richer insight into how eye dominance varies over visual space.^4^ To validate that this new method evokes interocular conflict in a way similar to conventional bCFS, I also ran a parallel experiment (performed by the same observers) applying traditional bCFS. I tested four predictions:

1. Sensory eye dominance varies over the visual field.
2. Sensory eye dominance assessed by tracking continuous flash suppression (tCFS) correlates with that assessed by bCFS.
3. The variation over space contains idiosyncratic features (the variation is observer-specific).
4. The variation over space contains a general signature (a nasal bias).

## Method

### Observers

Thirty-seven observers participated in the experiments (34 women, three men) as part of getting course credits for the psychology bachelor program in Utrecht. Ages ranged between 19 and 25 years (*mean* = 21.1; *sd* = 1.8 years) and 32 were right-handed. Besides having no stereovision (tested with the TNO test for stereovision (Walraven, 1971)), other exclusion criteria were colorblindness and no normal or corrected-to-normal vision. Informed consent was obtained before the experiments, and the experiments were approved by the local ethics committee of the faculty of social and behavioral sciences of Utrecht University (filed under 23-1920). One observer's data was excluded from the analysis because of an error in the saving of the data.

### Apparatus

The experiments were conducted using a custom-made stereoscope projecting images from two 23-inch Dell monitors (1920 × 1080-pixel resolution, 60 Hz refresh rate) in a dark room. Observers' heads were stabilized by a chin rest. Each mirror was oriented by an angle of 45° with respect to the viewer and one monitor. The middle of each monitor (i.e., the point of fixation) was placed perpendicular to the line of sight of the viewer. The viewing distance to the displays was 55 cm. The visual stimuli were presented using Matlab (R2022b update 5) and the Psychtoolbox, using an Ubuntu 22.04 release.

### Stimuli

In all experiments, the background of the monitors was gray (72.4 cd m^−2^). To facilitate binocular fusion, a 1/f pattern (6° VA wide; entire height of the monitor) was presented in the middle of each monitor. No fusion pattern was presented 3° VA below and above fixation. The fixation cross (a cross with diagonal lines) spanned the entire width of the screen (see below).

In the bCFS experiment, the target was a vertically oriented sinewave grating (2 cycles/°) presented within a circular aperture (radius of 1° VA) that changed from zero to maximum Michelson contrast in 3.54 seconds (Figure 1). The mask was a 6° × 6° VA dynamic high contrast mask flickering at 10 Hz (see Gayet, Douw, van der Burg, Van der Stigchel, and Paffen (2020) for details). For the first 18 observers, the mask was presented at full contrast (100% Michelson); for the final 18 observers, the contrast was changed to 50% Michelson.^5^ The target and mask remained on the screen for a maximum of 12 seconds. The target and mask's centers were positioned at −10 (left), 0° and 10° (right) VA relative to the fixation cross. If the target was presented to the left eye, the mask would be presented to the right eye, and vice versa.

In the tCFS experiment, the target (same dimensions and spatial frequency as in bCFS) and mask (the same specifics as in the bCFS experiment) moved across the screen with a constant velocity of 1° VA/s. The target and mask's centers moved along one of two trajectories (counterbalanced within each observer): (1) From the middle of the screens (at fixation) to 10° VA left of fixation, then from this left position to 10° VA right of fixation, and finally back to the fixation cross; (2) From the middle of the screens (at fixation) to 10° VA right of fixation, then from this right position to 10° VA left of fixation, and finally back to the fixation cross. One entire cycle lasted 40 seconds.

### Procedure

The order of the bCFS and tCFS experiments was counterbalanced within the observer pool (observers with odd observer numbers started with bCFS; the rest with tCFS). For the bCFS part, an observer started with five bCFS practice trials, with target and mask presented at fixation. Next, the bCFS experiment was conducted with 60 trials in total. The 60 trials of the bCFS experiment included 10 trials per eye for the target (left or right eye) per location (−10°, 0°, and 10° VA). In the tCFS part, an observer would practice in three tCFS trials in which target and mask were presented at fixation only. The actual tCFS experiment consisted of 20 trials. The 20 trials included 10 trial per eye (target left or right eye).

A bCFS trial would progress as follows: upon pressing the spacebar, the fixation cross would change from orange to green (alerting the observer that the trial would start). One second later the mask was presented and the target would start increasing in intensity. Target and mask were presented for a maximum duration of 12 seconds or until a response was made. The observer was asked to indicate to press the enter key as soon as the target was detected. After this, the fixation cross would turn from green to red until the space bar was pressed to start the next trial.

A tCFS trial would start with an observer pressing the spacebar. The target and mask would then start moving left or right. The observer was instructed to, from this moment on, continuously press the left arrow key as long as the target was visible, and to continuously press the right arrow key as long as the target was invisible. At each monitor refresh, a left arrow keypress would decrease the intensity of the target (until minimum intensity), and a right arrow keypress would increase the intensity of the target (until maximum intensity). The observer was asked to perform this task for the duration of the entire trial. An entire session lasted between 45 and 60 minutes.

### Data analysis

For bCFS, I calculated the median RT for each location and eye the target was presented to for each observer. After this, an eye dominance index (EDI_bCFS_) for each position (−10°, 0°, and 10° VA) was calculated per observer by:

$$\begin{array}{c} {\mathrm{EDI}}_{\mathrm{bCFS}}={\mathrm{RT}}_{l}/\left({\mathrm{RT}}_{l}+{\mathrm{RT}}_{r}\right) \end{array}$$

A value larger than 0.5 indicates right eye dominance; a value smaller than 0.5 indicates left eye dominance.

Some preprocessing was necessary to calculate eye dominance indices for the tCFS data: Each trial of an observer sampled the intensity needed to make the target visible or invisible along the horizontal meridian. The data of 10 trials per observer per eye (during which the target moved from the center to the left (or right), to the right (or left), and back to the center) were reorganized in 20 traces, where each trace contained target intensity from −10° VA to 10° VA along the horizontal meridian, for that eye. In the end, this led to each position along the axis being sampled 20 times per eye. To calculate the eye dominance index for tCFS (EDI_tCFS_), the intensity at each position for the left eye was divided by the sum of the intensities of the left and right eye:

$$\begin{array}{c} \mathrm{ED}I_{\mathrm{tCFS}}=I_{l}/\left(I_{l}+I_{r}\right). \end{array}$$

A value larger than 0.5 indicates right eye dominance; a value smaller than 0.5 indicates left eye dominance.

For the analysis across observers, the intensity was calculated by averaging the 20 samplings per position per eye (resulting in a single, averaged intensity, per position, per eye). For the analysis within observers, I used the 20 intensities per position per eye.

## Results

**Figure 2.**
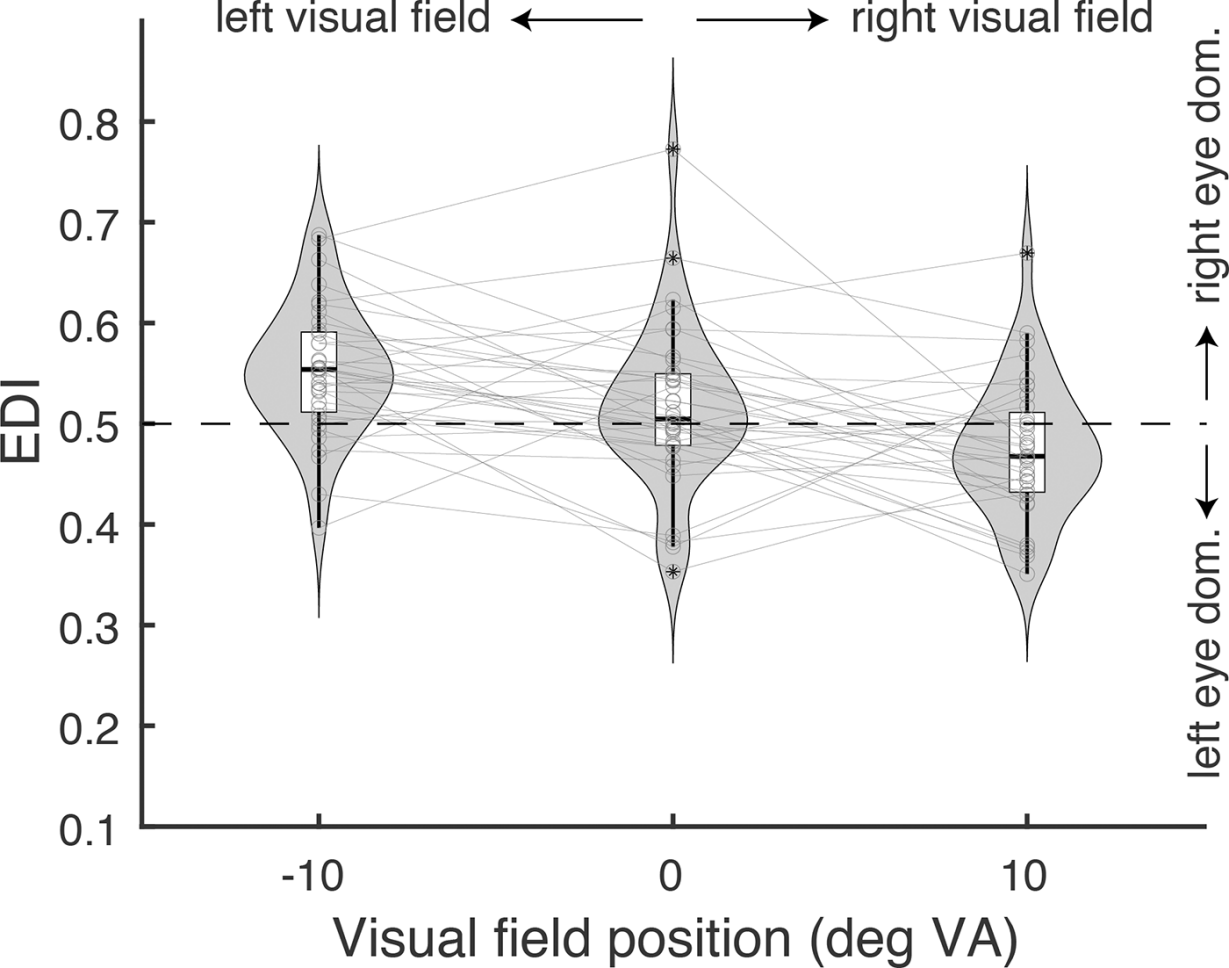
Results of the bCFS experiment: SED expressed as EDI_bCFS_ for three positions. A value larger than 0.5 indicates right eye dominance; a value smaller than 0.5 indicates left eye dominance. The EDI being larger than 0.5 for the left position (–10°) indicates right eye dominance; the EDI being smaller than 0.5 for the right position (10°) indicates left eye dominance: a nasal bias for both positions.

I first report the bCFS results to test whether the nasal bias reported by Sahakian et al. (2022) replicates (Figure 2). A repeated measures ANOVA shows that sensory eye dominance (quantified as the EDI_bCFS_) varied significantly across the three positions of the visual field (*F*(2,70) = 12.7, *p* < 0.001). Post hoc tests (Bonferroni corrected) further reveal that the EDI_bCFS_ for the left position was significantly larger than that of the middle position and the right position (*t*(35) = 2.65, *p =* 0.036; *t*(35) = 4.63, *p* < 0.001), and that the EDI_bCFS_ for the middle position was significantly larger than that for the right position (*t*(35) = 2.64, *p* < 0.05). To test whether an eye was dominant over the other, *t*-tests compared the EDI_bCFS_ for left, right and middle against a value of 0.5. The EDI_bCFS_ for the left position was significantly larger than 0.5 (*t*(35) = 4.88, *p* < 0.001), the EDI_bCFS_ for the middle position was not significantly different from 0.5 (*t*(35) = 1.211, *p* = 0.23), and the EDI_bCFS_ for the right position was significantly smaller than 0.5 (*t*(35) = −2.22, *p* < 0.05). These results support the nasal bias reported by Sahakian et al. (2022): the right eye was dominant when the target was presented to the left side of fixation (the nasal visual field for the right eye); the left eye was dominant when the target was presented to the right side of fixation (the nasal side for the left eye).

**Figure 3.**
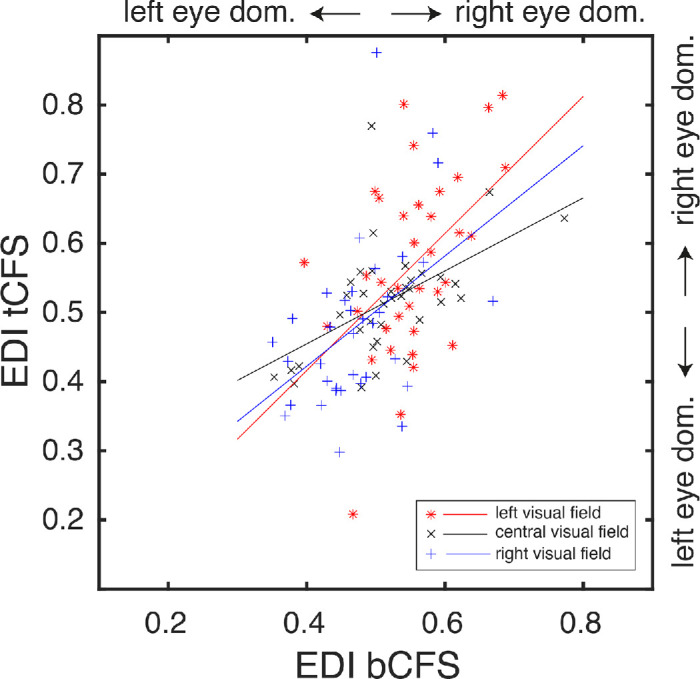
The relation between the EDI as assessed by bCFS (x-axis) and the EDI assessed by tCFS (y-axis). The red stars and the red line indicate the EDIs for –10° VA and the linear relation between them respectively; the black crosses and the black line indicate the EDIs for 0° VA and the linear relation between them respectively; the blue plusses and the blue line indicate the EDIs for 10° VA and the linear relation between them respectively.

Before delving into the results of the tCFS experiment, it first needs to be established that tCFS targets sensory eye dominance in a manner similar to bCFS. For this, I calculated the correlation between the EDI_bCFS_ and the EDI_tCFS_. Because the EDI_bCFS_ was measured for three positions (−10°, 0°, and 10° VA), and the EDI_tCFS_ continuously from −10° to 10° VA, I selected the EDI_tCFS_ for −10°, 0°, and 10° VA from the tCFS data. Figure 3 shows the correlation between the EDI_bCFS_ and EDI_tCFS_ for these three positions. Positive Pearson correlations indicate high correspondence between the EDI measured via bCFS and tCFS (for −10° VA: *r* = 0.49; *p* < 0.005; for 0° VA: *r* = 0.54; *p* < 0.001; for 10° VA; *r* = 0.45; *p* < 0.01). Thus the EDI assessed by bCFS and that assessed by tCFS were highly related. This validates the assumption that bCFS targets the EDI in a similar way as tCFS.

**Figure 4.**
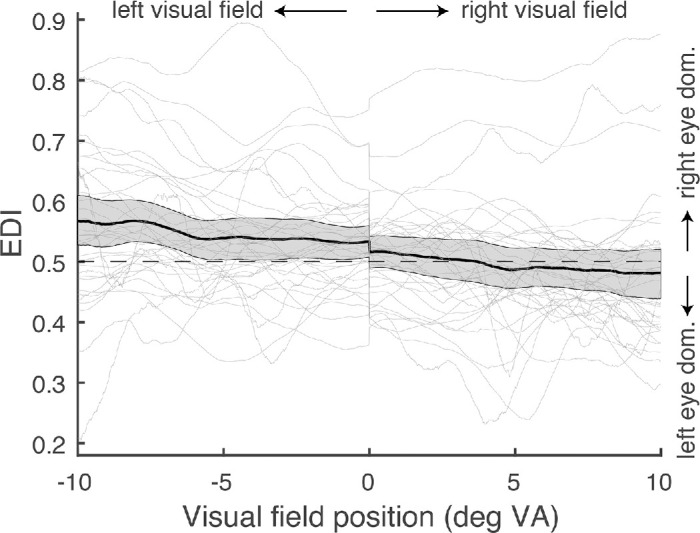
The tCFS results. The thin gray lines are EDIs for individual observers, the dark line the average EDI and the gray band the 95% CI. The negative slope indicates that there is a gradual change from right eye dominance for the left visual field to left eye dominance for the right visual field.

The next step in the analysis was quantifying EDI_tCFS_ on the group level: I performed a linear regression on the EDI_tCFS_ of the group (the black solid line in Figure 4): EDI_tCFS_ = x * B1 + B2 (where x is the location in space). With this equation, the nasal bias (I will refer to this now as the “hemifield bias”), as well as a general eye dominance (dubbed “eye bias” from now) can be quantified: if the slope (B1) is negative, SED at the left position of the visual field is larger for the right than for the left eye (indicating a nasal bias); if this slope is positive, eye dominance at the left position of the visual field is smaller for the right than for the left eye (indicating a temporal bias). The general SED is indicated by the offset (B2): if larger than 0.5, the right eye is generally more dominant (across the horizontal meridian); if smaller than 0.5, the left eye is generally more dominant. The result of this regression leads to a value of the slope (B1) of −0.048 (reflecting a 0.048 decrease in EDI per degree VA), and a value of the offset (B2) of 0.52. To find out about the significance, I tested whether the offsets of all observers were significantly different from 0.5. This was not the case (*t*(35) = 1.53, *p* = 0.14). The significance of the slopes was tested by testing whether the slopes were different from 0. The latter *was* the case (*t*(35) = −3.33, *p* < 0.01). The fact that the slope is negative shows that the EDI decreased with increasing degree VA. Again, this result supports a nasal bias in eye dominance: the right eye was more dominant at the left position; the left eye was more dominant at the right position.

**Figure 5.**
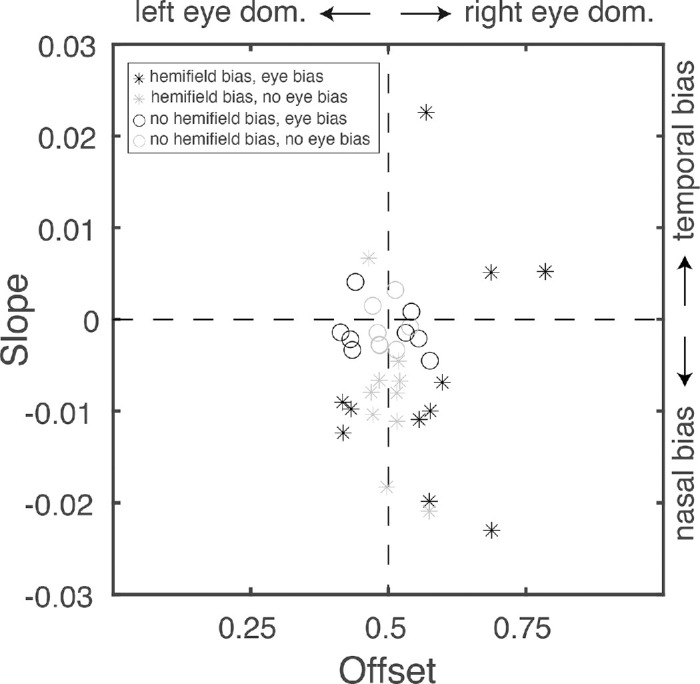
Slopes (indicating a hemifield bias) and offsets (referring to eye bias) for the observers. A black marker (circles and stars) indicates that the offset was significantly different from 0.5. A star indicates that the slope was significantly different from 0; a circle indicates it wasn't. A slope smaller than zero indicates a nasal bias; a slope larger than zero indicates a temporal bias. An offset smaller than 0.5 indicates left eye dominance; an offset larger than 0.5 indicates right eye dominance.

Next, the data was analyzed for each observer. I calculated the hemifield bias (slope) and eye bias (offset) for each observer and tested whether they were different from 0.5 and 0 respectively (Figure 5). The results of this analysis reveal that 19 out of 36 observers had a significant eye bias (and thereby significant left- or right-eye dominance): seven of those had an offset smaller than 0.5 and therefore classified as left-eye dominant; 12 of them had an offset larger than 0.5 and classified as right-eye dominant. Furthermore, 21 observers had a significant slope (and thereby a significant hemifield bias): four of those had a significant positive slope and thereby a temporal bias; 17 of those had a significant negative slope and thereby a nasal bias.

**Figure 6.**
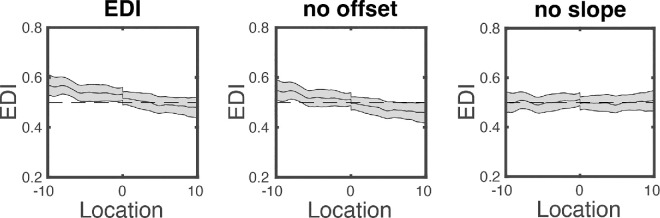
To assess local variations on top of the nasal effect, I removed both the offset (middle panel) and slope (right panel) of the EDI data (left panel).

Finally, as mentioned in the Introduction, it was expected that EDI would also vary *within* an observer. Within our paradigm, this would be reflected by local variations in EDI along the horizontal axis (from −10° to 10° VA). To quantify this local variation (which I will refer to as local bias), I removed both the slope and offset from the EDI_tCFS_ profile. Figure 6 shows the result of this procedure for the group data.

To assess whether there was a local bias in eye dominance on top of the general eye dominance and nasal effect, I tested whether there were locations in space for which the EDI was significantly different from 0.5 (after slope and offset were removed). For this, I used a custom-built cluster analyses (see Appendix B). In short, this analysis amounts to the following: for each datapoint (that is, a sampled location along the horizontal axis), a *t*-value was calculated from the individual observations (for observers the 20 trials; for the group data the 36 means), resulting in 1200 (i.e., the number of horizontal positions) *t*-values. To assess significance, and to minimize type 2 errors, a Monte Carlo simulation was applied in which, for every observation (20 per observer; 36 for the group analysis), the Fourier spectrum of the time series was taken and used to make a new time series containing the same power spectrum with scrambled phase. Thus, for every trial, a distribution was made containing the same shape of peaks in the EDI, but at random positions. From the resulting time series (20 or 36), a *t*-distribution was calculated. To get a null-distribution, this procedure (20 or 36 times making a new distribution and calculating the t-values) was repeated 10,000 times. For the following steps, the general permutation procedure of Maris and Oostenveld (2007) was used (again, see Appendix B).

**Figure 7.**
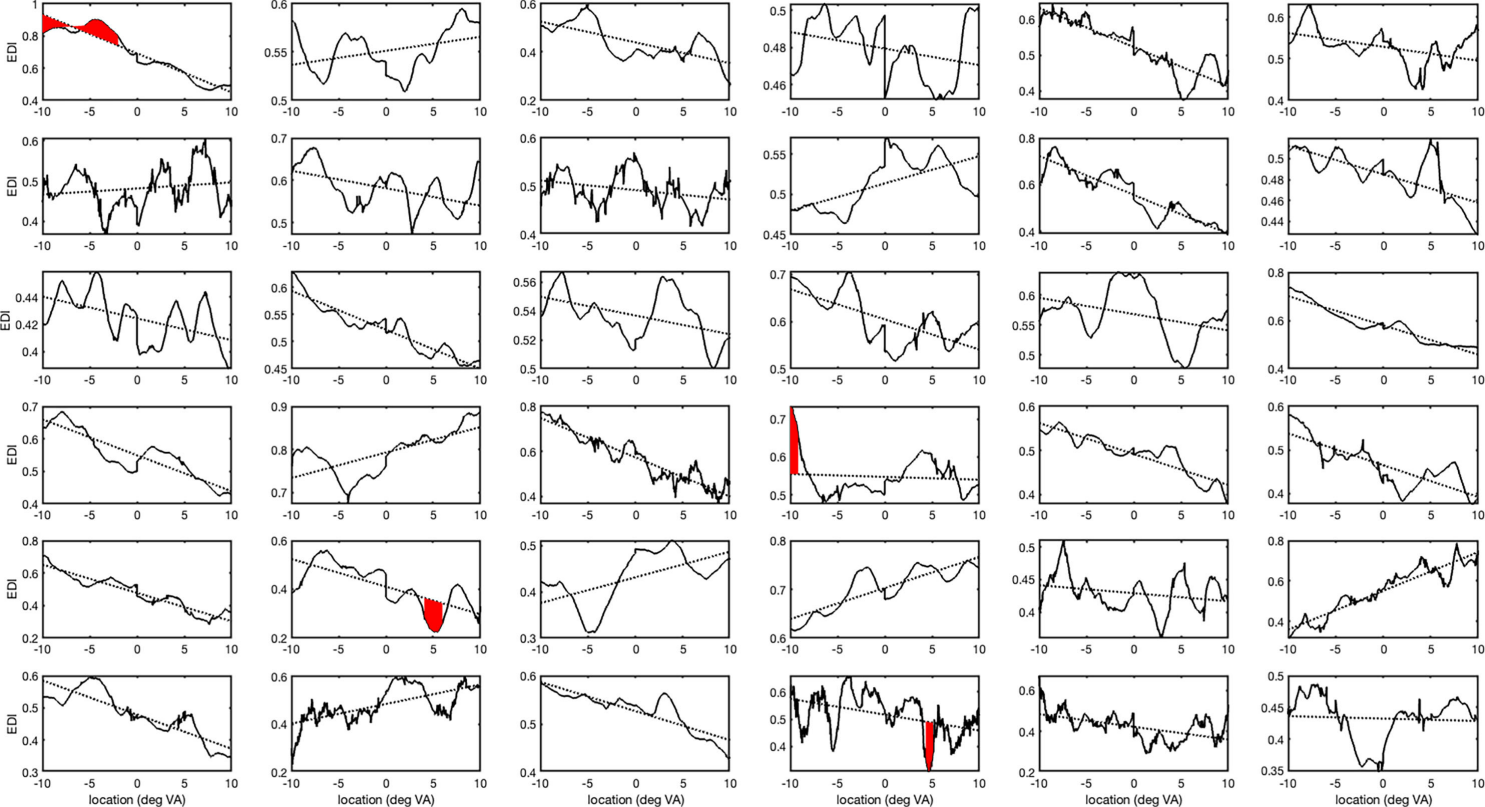
EDI-distributions (black solid line) for 36 observers. The individual EDI-distributions were calculated using the procedure outlined in Data Analysis. The dotted line displays the hemifield bias (negative: nasal bias; positive: temporal bias) and eye bias (an offset larger than 0.5: right eye dominance; and offset smaller than 0.5: left eye dominance). Red filled areas indicate locations where the EDI varied on top of the hemifield and eye bias.

Unsurprisingly, the above procedure did not reveal any local variations in EDI for the group data (Figure 6, right panel). For the individual observers, this analysis reveals that four out of the 36 observers had significant local bias zones on top of the eye and hemifield bias (Figure 7). These zones are indicated with the red filled area in Figure 7.

## Discussion

In this study I set out to quantify both generic (across observers) and idiosyncratic (observer-specific) aspects of SED. The results show that, across observers, there was a generic hemifield bias reflected by the finding that for the left visual field, the SED for the right eye was larger, and for the right visual field, the SED for the left eye was larger. Because right SED for the left visual field and left SED for the right visual field correspond to a bias for the nasal visual field over the temporal one for both, these results replicate findings showing that, during interocular conflict, information shown in the nasal visual field receives a processing advantage over information shown to the temporal visual field. On top of this general hemifield effect, three other aspects of SED were observed. First, half of the observers could be classified as having a location-independent preference for the left or right eye (the SED was biased toward the left or right eye). Second, although most observers were classified as having a nasal hemifield bias, a small subset (four out of 36) showed a temporal hemifield bias. Third, a few observers’ results displayed local variations in SED (on top of a general eye and hemifield bias): confined regions in space where one eye dominated over the other. Taken together, these results show that SED across visual space can be quantified by three factors: (1) a hemifield bias, (2) an eye bias, and (3) a local bias. The first factor leans toward a preference for the nasal visual field (where SED is dependent on the location left or right of fixation), whereas the two other factors will differ between observers: from left to unbiased to right eye dominance for SED and from an absence of local variation to (potentially) local zones in which SED differs independently of the hemifield and general eye bias.

The generic nasal advantage on the group level reported here is in line with that reported by Sahakian et al. (2022) and Mustonen et al. (2018) who used bCFS to assess a visual field effect in interocular competition. However, as noted before, this does not warrant the conclusion that there is a general nasal advantage during interocular conflict across the *entire* visual field, since while there are other studies also reporting a nasal preference (Chen & He, 2003; Kaushall, 1975), still other studies report a temporal preference (Crovitz & Lipscomb, 1963; Fahle, 1987; Stanley et al., 2011; Stanley et al., 2019), and still other find none (Dieter et al., 2017b). Interestingly, previous interpretations of the reported nasal-temporal asymmetries potentially offer a way out of the apparent conflicting conclusions. For example, as Fahle (1987) already noted, in the far periphery of the visual field, a temporal bias during interocular conflict correlates with a sharper decrease in hyperacuity for the nasal versus temporal visual hemifield (Fahle & Schmid, 1988). Fahle related these results to a greater amount of projections from the nasal hemiretina (compared to the temporal hemiretina) to the visual cortex of the macaque (LeVay, Connolly, Houde, & van Essen, 1985). Additionally, neurophysiological results show that the nasal hemiretina contains 40 to 50% higher rod and cone density (Curcio, Sloan, Kalina, & Hendrickson, 1990) and a three times higher ganglion cell density (Curcio & Allen, 1990). For the far visual periphery then, a bias for the temporal visual hemifield during interocular conflict (i.e. the result of Fahle (1987)) appears to go hand in hand with a neurophysiology supporting this. Could a nasal preference be restricted to the nearer visual periphery? This appears to be the case, it has been noted that a nasal visual field advantage is adaptive when confronted with monocular occlusion zones (Arnold, 2011; Nakayama & Shimojo, 1990; Sahakian et al., 2022). As Sahakian et al. (2022) demonstrated, when fixating a distant object that is partly occluded by a nearby object, the fixated part of the object of interest is in the nasal visual field, while the occluding part of the *nearby* object (the occluder) is in the temporal visual field. Because the fixated object is the object of interest, it is adaptive to bias perception toward the nasal information, at the cost of processing the temporal information. I therefore suggest that a nasal bias is adaptive in areas surrounding the central visual field, whereas a temporal advantage is adaptive in the far periphery. Future studies could test this hypothesis by applying tCFS also in the far periphery. Still, opposing this hypothesis is the fact that Stanley et al. (2011) and Stanley et al. (2019) reported a *temporal* bias during interocular conflict for targets presented at 4° VA and 0.38°–1.13° VA, respectively. Inspecting their results, however, reveals that the number of observers they used is limited (5 and 9, respectively). Notably, in the current study, eight of the 36 observers showed a tendency for a temporal advantage. Thus it is conceivable that the differences in naso-temporal asymmetries are (partly) the result of a sampling bias. Also, based on the fact that different studies used different methods to evoke interocular conflict (the results of Crovitz and Lipscomb (1963), Stanley et al. (2011) and Stanley et al. (2019) were acquired using onset rivalry, those of Chen and He (2003), Kaushall (1975), and Fahle (1987) were acquired using ongoing binocular rivalry, and those by Mustonen et al. (2018), Sahakian et al. (2022) and the ones reported here were acquired using (a variant of) bCFS), future studies need to compare nasal-temporal asymmetries with different variants (e.g., onset rivalry, bCFS, ongoing) of interocular conflict.

The conclusion of local biases in eye dominance is supported by several studies also reporting idiosyncratic variations within observers (Dieter et al., 2017b; Stanley et al., 2011; Stanley et al., 2019; Xu et al., 2011). I speculate here that two basic underlying mechanisms, or a combination of them, could give rise to local idiosyncratic biases (also see Xu et al. (2011)): (1) a difference in monocular processing of each eye's input, or (2) an imbalance in the competition between the inputs. In the first possibility, the strength of one eye's monocular input to the competition is different, for example as a result of a localized microlesion in a lens or retina, or as a result of altered (local/retinotopic) processing in primary visual cortex. In the second possibility, the competition is locally biased in favor of one eye's input. These mechanisms are reflected in pioneering (Blake, 1989) and contemporary (Li, Rankin, Rinzel, Carrasco, & Heeger, 2017) neural models of binocular rivalry, which involve a stage where information is represented monocularly, as well as a stage at which the information competes (e.g., “opponency neurons” in Li et al., 2017). Interestingly, Xu et al. (2011) speculated that the second proposed mechanism was more likely to be responsible for the local biases they reported: in their study, local biases in SED did not correlate well with local monocular contrast thresholds. However, (Fahle, 1987; Fahle, Edelman, & Poggio, 1995; Fahle & Schmid, 1988) showed that the nasal-temporal imbalances in SED correlated with monocular hyperacuity estimates, suggesting that the spatial bias *did* originate from differing monocular input.

How did the newly adapted method—tCFS—perform in assessing sensory eye dominance? The fact that I observed high correspondence between EDI's assessed by bCFS and tCFS supports the conclusion that tCFS is a valid method to assess (local) SED. It therefore seems to fit the purpose of assessing SED continuously and relatively quickly. However, the method, as applied here, has a potential shortcoming: the intensity, indicated by an observer through key presses, of the target presented at location X, is highly dependent on the intensity at X-1. The reason for this is simply that the observer only had the option to gradually, opposed to abruptly, adjust the intensity of the target. It is therefore by definition the case that the intensities at different locations are not independent: intensities (and in the end, EDI's) for locations adjacent to a certain location in space at least partly reflect what was perceived at that certain location in space. This dependency on responses to adjacent locations limits the method's potential to detect fine-grained differences in local EDIs. This limitation is also dependent on the speed at which the target moves: a lower speed makes finding fine-grained differences more likely (but the experiment more time-consuming); a higher speed will make this less likely (but more economically, timewise). Note that this observation motivated my choice for the design of a null-distribution for local imbalances: the null-distributions reflect gradual transitions in EDI (i.e. spatial dependencies) while removing biases fixed to certain locations. The dependency of the local EDIs on responses to neighboring positions might also explain why, at first sight, the current study reveals less observer-specific (i.e., idiosyncratic) local biases compared to those reported in earlier ones (Carter & Cavanagh, 2007; Dieter, Sy, & Blake, 2017a; Dieter, Sy, & Blake, 2017b; Stanley et al., 2011; Stanley et al., 2019). On the other hand, the idiosyncratic biases reported in those studies were reported without removing general overall eye biases (e.g., the left eye being generally more dominance than the right, independent of location) and hemifield biases (e.g. a nasal or temporal bias within an observer), meaning that the biases reported in those studies likely included these two kinds of general biases.

A shortcoming of the study is that I did not measure eye movements. I therefore cannot claim that, during tCFS tracking, observers refrained from moving their eyes. I am, however, confident that eye movements did not systematically affect the results reported. For one, the most likely eye movement pattern conceivable would be for an observer to move their eyes to the target (and mask). However, it is known that when observers move their gaze to the visual periphery, eye dominance shifts in line with the side the gaze is directed to: when gazing to the left, eye dominance shifts towards more left-eye dominance; when gazing to the right, eye dominance shifts toward more right-eye dominance (Carey & Hutchinson, 2013; Khan & Crawford, 2001), which of course amounts to a temporal bias. This fact makes it difficult to reconcile the nasal bias with an eye movement account, suggesting that if eye movements were made in line with the movement, effects might be underestimated rather than overestimated.

To conclude, I here applied a novel method—tCFS—to assess variations in SED across the visual field: the method shows to be a fast and effective way to assess SED continuously. The method reveals that SED across the visual field can be described by generic and subject-dependent, idiosyncratic biases: Within the visual field tested (0° to 10° VA of the horizontal axis), I observed a general nasal visual field bias for the sample tested. In addition, I described participant-specific variations in EDI as (1) a general preference for using the left, right, or either eye and (2) a bias towards the temporal or nasal hemifield and (3) idiosyncratic local biases on top of the hemifield and general eye preference.

## Footnotes

1 Please note that, as explained above, a nasal preference during interocular competition corresponds to the right eye being dominant at the left visual field, and the left eye at the right visual field. For a temporal bias, the relation between eye dominance and visual field is reversed.

2 Six datasets in Sahakian et al. (2022) and one in Mustonen et al. (2018).

3 Although the method used here is different from that of Alais et al. (2024) I chose to use the same name as they introduced: tracking continuous flash suppression.

4 The continuous aspect also makes it superior to onset rivalry, where different locations in visual space can only be probed at separate time points.

5 We combined the datasets to increase statistical power. However, we also performed all the analyses separately, for the group with the 100% mask contrast and the group with the 50% mask contrast (Appendix A).

## Appendix A

I split the analysis for the two groups that performed the bCFS and tCFS experiment with two different Michelson contrast levels of the dynamic mask.

### Results when the mask contrast was 100% (first 18 observers)

For the bCFS-experiment, a repeated measures ANOVA revealed that sensory eye dominance (quantified as the EDIbCFS) did not vary significantly across the three positions of the visual field (*F*(2,34) = 2.75, *p* = 0.08). Targeted analyses showed that there was evidence for one eye being more dominant than the other: the EDIbCFS for the left position was significantly larger than 0.5 (*t*(17) = 2.16, *p* < 0.001), although the EDIbCFS for the middle position was not significantly different from 0.5 (*t*(17) = 0.41, *p* = 0.69), nor was the EDIbCFS for the right position (*t*(17) = −1.54, *p* = 0.08).

The correlations between EDIs assessed by bCFS and tCFS revealed that the correlation between EDIbCFS and EDItCFS were significant for the left and the middle position, but not for the right (for −10° VA: *r* = 0.55; *p* < 0.05; for 0° VA: *r* = 0.73; *p* < 0.001; for 10° VA; *r* = 0.20; *p* = 0.42).

The slope (B1) of the tCFS data was −0.057 (reflecting a 0.057 decrease in EDI per degree VA), and the offset (B2) was 0.53. The offset of the 18 observers did not differ significantly different from 0.5 (*t*(17) = 1.84, *p* = 0.08). The slope *was* significantly different from 0 (*t*(17) = −3.43, *p* < 0.01). Performing the cluster analysis on the data of the first 18 observers did not reveal any local EDI clusters for the group.

### Results when the mask contrast was 50% (last 18 observers)

A repeated measures ANOVA on the bCFS data of the last 18 observers showed that sensory eye dominance (quantified as the EDIbCFS) varied significantly across the three positions of the visual field (*F*(2,34) = 12.57, *p* < 0.001). Post hoc tests (Bonferroni corrected) further reveal that the EDIbCFS for the right position was significantly smaller than that of the left position and the middle position (*t*(17) = −4.35, *p* < 0.01; *t*(17) = −3.35, *p* < 0.05). The EDIbCFS for the left position was not significantly different from that for the middle position (*t*(17) = 2.26, *p* = 0.11).

The EDIbCFS for the left position was significantly larger than 0.5 (*t*(17) = 5.31, *p* < 0.001), the EDIbCFS for the middle position was not significantly different from 0.5 (*t*(17) = 1.43, *p* = 0.17), and the EDIbCFS for the right position was not significantly smaller than 0.5 (*t*(17) = −1.67, *p* = 0.06).

The correlations between EDIs assessed by bCFS and tCFS revealed significant correlations for the left and right position, but not for the middle one (for −10° VA: *r* = 0.55; *p* < 0.05; for 0° VA: *r* = 0.44; *p* = 0.07; for 10° VA; *r* = 0.55; *p* < 0.05).

The slope (B1) of the tCFS data was −0.0039 (reflecting a 0.0039 decrease in EDI per degree VA), the offset (B2) was 0.51. The offset was not significantly different from 0.5 (*t*(17) = 0.62, *p* = 0.55). The slope was not different from 0 (*t*(17) = −1.62, *p* = 0.12).

Together, the separate analyses show that the results for both mask-contrast settings were qualitatively, but not quantitatively similar. I suggest that the differences between the separate analyses, and between these and the analyses of the complete dataset are due to a difference in experimental power.

## Appendix B

To find whether local imbalances in EDI could be observed on top of a general eye and hemifield bias,I undertook the following analysis steps. First for the group:

1. Both the slope (−0.048) and the offset (0.52) of the EDIs of 1200 (locations) × 36 (observers) data points were removed.
2. 1200 *t*-values were calculated for the 1200 EDIs.
3. Clusters were defined for which neighboring *t*-values were larger than the critical *t*-value (2.03 in this case). The *t*-values were summed for continuing, significant *t*-values: these are the summed *t*-values for clusters of EDIs different from 0.
4. After adding an offset of 1 (because Fourier analysis cannot deal with negative values), the Fourier spectrum was determined for each horizontal sampling of EDIs. Next, an EDI was recreated with an inverse Fourier transform, with random phase, for each of the 36 horizontal samplings. After this, the offset of 1 was removed again for each of the 36 recreated series.
5. 1200 t-values were calculated for the recreated EDIs.
6. Clusters were defined for which neighboring *t*-values were larger than the critical *t*-value (2.03 in this case). The *t*-values were summed for continuing, significant *t*-values: these are the summed *t*-values for clusters of recreated EDIs different from 0.
7. The largest of the summed *t*-values of the recreated EDIs was determined.
8. Steps 4–7 were repeated 10,000 times, saving the largest summed *t*-value of each iteration.
9. To determine the *p* value of clusters of actual EDIs exceeding “chance,” the fraction of summed *t*-values larger than the 10,000 generated *t*-values was calculated. The *p* value then is 1 minus that fraction. Thus a *p* value = 0.05 would mean that 95% of the summed *t*-values of the actual data exceeded those of the generated data.
10. A significant, local imbalance in EDI was now defined as a cluster having a *p* value < 0.05.

For the analysis of individual observers, the 20 trials per observer were used in the above analysis (instead of the 36 average EDIs of the observers).
